# PKLM: A Flexible MCAR Test Using Classification

**DOI:** 10.1017/psy.2024.14

**Published:** 2025-01-03

**Authors:** Meta-Lina Spohn, Jeffrey Näf, Loris Michel, Nicolai Meinshausen

**Affiliations:** 1 ETH Zürich, Seminar for Statistics, Zürich, Switzerland; 2 Inria PreMeDICaL Team, Montpellier, France; 3 QuantCo, Zürich, Switzerland

**Keywords:** random projections, tree ensembles, random forest, KL-divergence, permutation

## Abstract

We develop a fully nonparametric, easy-to-use, and powerful test for the missing completely at random (MCAR) assumption on the missingness mechanism of a dataset. The test compares distributions of different missing patterns on random projections in the variable space of the data. The distributional differences are measured with the Kullback-Leibler Divergence, using probability Random Forests (Malley et al., 2011). We thus refer to it as “Projected Kullback–Leibler MCAR” (PKLM) test. The use of random projections makes it applicable even if very few or no fully observed observations are available or if the number of dimensions is large. An efficient permutation approach guarantees the level for any finite sample size, resolving a major shortcoming of most other available tests. Moreover, the test can be used on both discrete and continuous data. We show empirically on a range of simulated data distributions and real datasets that our test has consistently high power and is able to avoid inflated type-I errors. Finally, we provide an R-package PKLMtest with an implementation of our test.

## 1 Introduction

Dealing with missing values is an integral part of modern statistical analysis. In particular, the assumed mechanism leading to the missing values is of great importance. Based on the work of Rubin (1976), there are three groups of missingness mechanisms usually considered: The values may be missing completely at random (MCAR), meaning the probability of a value being missing does not depend on the observed or unobserved data. In contrast, the probability of being missing could depend on observed values (missing at random, MAR) or on unobserved values (missing not at random, MNAR).

As stated in Yuan et al. (2018), “a formal confirmation of the MCAR missing data mechanism is of great interest, simply because essentially all methods can still yield consistent estimates under MCAR even if the underlying population distribution is unknown”. While there is, at least for imputation, a number of approaches that can deal with a MAR missing data mechanism such as multivariate imputation by chained equations (mice) (Buuren & Groothuis-Oudshoorn, 2011; Deng et al., 2016), many commonly used methods explicitly rely on the validity of the MCAR assumption. Examples are the easy-to-use listwise-deletion and mean-imputation methods (Little & Rubin, 1986). Consequently, the original paper on MCAR testing (Little, 1988) has been cited close to 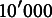 times according to google scholar. Recent papers (involving psychometric analysis) that test the MCAR assumption in order to justify listwise-deletion include Brown et al. (2020), Charles et al. (2021), Hawes et al. (2021), Sun and Chen (2022), de Vos et al. (2022), Rajeb et al. (2023), Zarate et al. (2023), and Langer et al. (2024). As such, it is important to reliably test the MCAR assumption.

The testing framework is of an ANOVA-type: when observing a dataset with missing values, there are *n* observations and *G* missingness patterns, 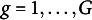 . The observations belonging to the missingness pattern *g* can be seen as a group, such that we observe *G* groups of observations. The MCAR hypothesis now implies that the distribution of the observed data in all groups is the same, while under the alternative at least two differ. This is technically testing the observed at random (OAR) assumption defined in Rhoads (2012), see also the end of Section 3 for a discussion. This distinction can be avoided by assuming the missingness mechanism is MAR, which is what is usually implicitly done (Li & Yu, 2015).

The idea of testing the MCAR assumption traces back to Little (1988). While some more refined versions of this testing idea were developed since then (Chen & Little, 1999; Jamshidian & Jalal, 2010; Kim & Bentler, 2002), there has not been a lot of progress on distribution-free MCAR tests, able to detect general distributional differences between the missingness patterns. Li and Yu (2015) recently made a step in that direction. Their test is completely nonparametric and shown to be consistent. Empirically it is shown to keep the level and to have a high power over a wide range of distributions. An application area where their proposed test struggles is for higher-dimensional data with little or no complete observations. Their testing paradigm is based on “a reasonable amount” of complete cases and all pairwise comparisons between the observed parts of two missingness pattern groups. This is problematic, since, as the dimension *p* increases, the number of distinct patterns *G* tends to grow quickly as well. The most extreme case occurs when 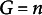 , that is, every observation forms a missingness pattern group on its own. Consequently, their test appears computationally prohibitively expensive for 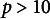 . Additionally, as the dimension increases, both the number of complete cases and the number of observations per pattern tend to decrease, both contributing to a reduction in power for the test in Li and Yu (2015).

In this paper, we try to circumvent these problems in a data-efficient way, by employing a one v.s. all-others approach and using *random projections* in the variable space. Considering observations that are projected into a lower-dimensional space allows us to recover more complete cases. As realized by Li and Yu (2015), the problem of MCAR testing, as described above, is a problem of testing whether distributions across missingness patterns are different. The method presented here relies on some of the core ideas of Näf et al. (2023) and Cai et al. (2020), who do distributional testing using classifiers. We extend the ideas of Cai et al. (2020) to be usable for multiclass classification and use the projection idea of Näf et al. (2023) to build a test that is usable and powerful even for high dimensions. Moreover, using a permutation approach, we are able to provably keep the nominal level 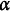 for all *n*. As outlined later, this is in contrast to other tests, for which the level might be kept only asymptotically, or is even unclear. The approach of random projections together with a permutation test also allows to extract more information than just a global hypothesis test. We make use of this to calculate individual *p*-values for each variable. Such a partial test for a variable addresses the null hypothesis that, once that variable is removed, the data is MCAR. Together with the test of overall MCAR, this might point toward the potential source of deviation from the null, that is, the variables causing an MCAR violation.

The paper is structured in the following way. Section 2 introduces notation. Section 3 details the testing framework including the null and alternative hypotheses we consider. Section 4 then showcases how to perform this test in practice and details the algorithm. Section 5 shows some numerical comparisons for type-I error control and power. Section 6 explains the extension of partial *p*-values, while Section 7 concludes. Appendix A contains the proofs of all results, while Appendix B adds some additional details and shows computation times of the different tests.

### 1.1 Contributions

Our contributions can be summarized as follows: We develop the PKLM-test, an easy-to-use and powerful nonparametric test for MCAR, that is applicable even in high dimensions. We thereby extend the testing approach of Cai et al. (2020) to multiclass testing, which in connection with random projections in the variable space and the Random Forest classifier leads to a powerful test for both discrete and continuous types of data. To the best of your knowledge, no other test is as widely applicable and powerful. Moreover, we are able to formally prove the validity of our *p*-values for any sample size and number of groups *G*. As we demonstrate in our simulations, this is remarkable for the MCAR testing literature. It appears no other MCAR test has such a guarantee and many have inflated type-I errors, even in realistic cases, see e.g. the discussion in Jamshidian and Jalal (2010).

As an extension, we can compute partial *p*-values corresponding to each variable, addressing the question of the source of violation of MCAR among the variables. We demonstrate the validity and power of our test on a wide range of simulated and real datasets in conjunction with different MAR mechanisms. Finally, we make our test available through the R-package PKLMtest, available on https://github.com/missValTeam/PKLMtest and on CRAN.

### 1.2 Related work

Previous advances for tests of MCAR were mostly addressed by Little (1988) (referred to as “Little-test”) and extensions (Chen & Little, 1999; Kim & Bentler, 2002) under the assumption of joint Gaussianity. To the best of our knowledge, the only distribution-free tests are developed in Jamshidian and Jalal (2010), Li and Yu (2015) and Zhang et al. (2019). The first paper develops a test (referred to as “JJ-test”), which is distribution-free but is only able to spot differences in the covariance matrices between the different patterns. As such, the simulation study in Li and Yu (2015) shows that their test (referred to as “Q-test”), which can detect any potential difference, has much more power than the JJ-test. Moreover, the JJ-test requires prior imputation of missing values, which appears undesirable. Zhang et al. (2019) develop a test that can be used to subsequently also consistently estimate certain estimators under MCAR. Their test requires a set of fully observed “auxiliary” variables that can be used to first test and then estimate properties of some variable of interest. As such their approach and goals are quite different from ours.

Consequently, the test closest to ours is the fully nonparametric method in Li and Yu (2015). However, it is computationally costly or even infeasible to use their test with dimensions typically found in modern datasets 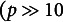 ), as all pairwise comparisons between missingness patterns are calculated. While this could in principle be avoided by only checking a subset of pairs, we empirically show that, even if all pairwise comparisons are performed, our test has comparable or even higher power than theirs in their own simulation setting. This gap only increases with the number of dimensions or with a decrease in the fraction of fully observed cases.

We also address a major issue in the MCAR testing literature: none of the proposed methods has a finite sample guarantee of producing valid *p*-values and for some it can even be empirically checked that the produced *p*-value is not valid in certain settings. If *Z* is a *p*-value generated from a statistical test, then it is valid if 
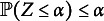
 under 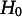 for all 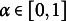 , see e.g., Lehmann and Romano (2005). Figure 2 in Section 5 shows some example of previous tests violating this validity of *p*-values. This issue might be surprising since the requirement of a valid *p*-value might be the most basic demand a statistical test needs to meet. For the Little-test, this is generally true under normality or asymptotically, that is if the number of observations is going to infinity, under some moment conditions and conditions on the group size. Despite this, Section 5 shows that type error rates can strongly exceed the desired level even in samples of 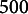 observations. The same holds for the JJ-test of Jamshidian and Jalal (2010) for which we sometimes observed a strong inflation of the level. As with the JJ-test, Li and Yu (2015) also do not provide a formal guarantee that the level is kept. Though in our own simulation study, which is similar to theirs, we did not find any notable violation of the level for their test.

To conduct our test, we adapt and partially extend the approaches of Cai et al. (2020) and Näf et al. (2023). The former develops a two-sample test using classification, an approach that has gained a lot of attention in recent years (see e.g., Kim et al. (2021) or Hediger et al. (2022) for a literature overview). We extend this approach to multiclass testing, to obtain a test statistic akin to Cai et al. (2020), but using the out of bag (OOB) probability estimate of the Random Forest (RF) instead of the in-sample probability. This was already hinted in Hediger et al. (2022) to increase the power of the two-sample testing approach designed by Cai et al. (2020). Näf et al. (2023), on the other hand, use random projections to increase the sample efficiency in the presence of missing values. This simple idea makes our test applicable and powerful, even in high dimensions, and even if the number of patterns *G* is the same as the number of observations. It can also provide additional information together with the rejection decision, as we demonstrate in Section 6. Finally, through an efficient permutation testing approach, we are able to formally guarantee that our test produces valid *p*-values for *any n* and any number of groups *G*. It appears that the PKLM-test is the first MCAR test with such a guarantee. Table 1 summarizes some of the properties of different tests. In particular, “mixed data types” refers to a possible combination of continuous data (such as income) and discrete data (such as gender), while “power beyond differences in first and second moments” means the test is able to detect differences between distributions, even if their means or variances are identical. Though this is difficult to show formally, it appears quite clear that the nonparametric nature of our approach allows for the detection of differences in distributions between patterns, even if the missingness groups all share the same mean or covariance matrix. As outlined in Yuan et al. (2018), this is crucial for the detection of general MCAR deviations and is not the case, for instance, for the widely used Little-test. Appendix C studies a simulated MAR example taken from Yuan et al. (2018), whereby observed means and variances are approximately the same across different groups. Tests such as the Little-test have no power in this example, yet with our approach, we reach a power of 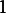 .

**Table 1:** Illustration of some of the properties of various tests

	PKLM	Q	Little	JJ
Computational complexity	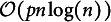	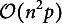	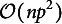	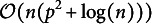
Can be used without	Yes	No	No	Yes
complete observations				
Mixed data types possible	Yes	No	No	No
Does not require initial imputation	Yes	Yes	Yes	No
Power beyond differences	Yes	Yes	No	No
in first and second moments				

*Note*: For details on the calculation of the computational complexities we refer to Appendix B.

## 2 Notation

We assume an underlying probability space 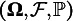 on which all random elements are defined. Along the lines of Muzellec et al. (2020), we introduce the following notation: let 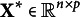 be a matrix of *n* complete samples from a distribution 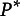 on 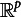 . We denote by 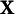 the corresponding incomplete dataset that is actually observed. Alongside 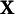 we observe the missingness matrix 
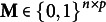
 , of which an entry 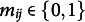 is 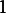 , if entry 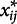 is missing, and 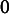 , if it is observed. Each unique combination in 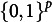 in 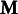 is referred to as a missingness pattern and we assume that there are 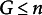 unique patterns in 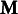 . As an example, for 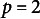 , we might have the pattern 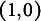 (first value missing, second observed), 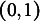 (first value observed, second missing) or 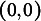 (both values are observed). We do not consider the completely missing pattern, in this case 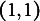 .

We assume that each row 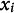 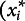 ) of 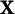 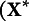 ) is a realization of an i.i.d. copy of the random vector *X* 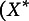 ) with distribution *P* 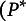 ). Similarly, *M* is the random vector in 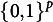 encoding the missingness pattern of *X*. Furthermore, we assume that *P* 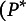 ) has a density *f* 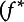 ) with respect to some dominating measure. For a random vector *X* or an observation *x* in 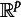 and subset 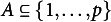 , we denote as 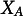 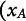 ) the projection onto that subset of indices. For instance if 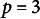 and 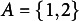 , then 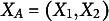 
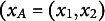
 ). For any set 
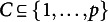
 , we denote by 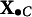 the matrix of *n* observations projected onto dimensions in *C*, so that 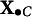 is of dimension 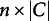 . Similarly, for 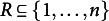 , 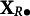 denotes the matrix of observations in set *R*, over all dimensions, so that the dimension of 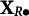 is given by 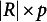 . We denote by 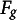 (respectively 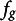 ) the complete distribution (density) of the data in the 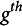 missingness pattern group. A quick overview of the notation including the use of indices for the number of missingness patterns, dimensions, observations, projections, and permutations is given in Table 2.

**Table 2:** **Notation**: Summary of the notation used throughout the paper, with (“partial”) and without (“full”) considering the missing values.

Notation	Partial	Full
Distribution	*P*	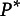
Dataset	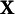	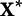
Observation in 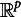	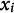	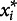
Random vector	*X*	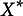
Density	*f*	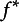
Number of missingness patterns	*G*	
Number of dimensions	*p*	
Number of observations	*n*	
Number of projections	*N*	
Number of permutations	*L*	

## 3 Testing framework

In this section, we formulate the specific null and alternative hypotheses for testing MCAR considered by the PKLM-test. Recalling the notation of Section 2, a missingness pattern is defined by a vector of length *p*, consisting of ones and zeros, indicating which of the *p* variables are missing in the given pattern. We divide the *n* observations into 
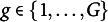
 unique groups, such that the observations of each group share the same missingness pattern. Each group 
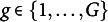
 contains 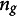 observations such that 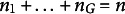 . Let 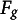 denote the joint distribution of the *p* variables in the missingness pattern group *g*, such that the 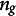 observations of the group *g* are i.i.d. draws from 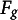 . As stated in Li and Yu (2015), testing MCAR can be formulated by the hypothesis testing problem (1)

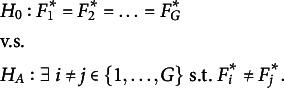



We want to emphasize the use of 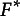 in the testing problem (1), indicating that these hypotheses involve distributions we cannot access. Thus, (1) needs to be weakened. Borrowing the notation of Li and Yu (2015), for missingness pattern group *g* we denote with 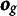 and 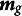 the subsets of 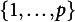 indicating which variables are observed and which are missing, respectively. We denote the induced distributions by 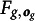 and 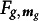 . For two groups *i* and *j*, we denote by 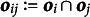 the shared observed variables of both groups. As mentioned in Li and Yu (2015), it is not possible to test (1) reliably, since the distribution 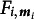 of the unobserved variables is inaccessible. Thus, Li and Yu (2015) consider the following hypothesis testing problem (2)

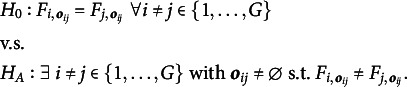



The null hypothesis 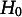 of (2) is implied by 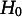 of (1), but not vice-versa. In other words, if we can reject the null hypothesis of (2), we can also reject the null hypothesis of (1). But if the null hypothesis of (2) cannot be rejected, there could still be a distributional change for different groups in the unobserved parts, so that the null hypothesis of (1) is not true. In this case, the missingness mechanism would be MNAR. Thus, using the terminology of Rhoads (2012), (2) tests the “observed at random” (OAR) hypothesis instead of the MCAR hypothesis. The differentiation can be circumvented by assuming that the missingness mechanism is MAR, which is the approach usually taken, see Li and Yu (2015).

The comparison of all pairs of missingness groups in the hypothesis testing problem (2) is problematic, however, as laid out in the introduction. In the following, we circumvent this problem in a data-efficient way, considering a one v.s. all-others approach and employing *random projections* in the variable space. Considering observations that are projected into a lower-dimensional space allows us to recover more complete cases. Let 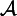 be the set of all possible subsets of 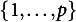 with at most 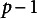 elements. For 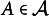 we define by 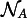 the indices in 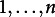 of observations that are observed with respect to projection *A*, i.e., observations of which the projection onto *A* is fully observed. These observations may belong to different missingness pattern groups 
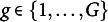
 . As an example, 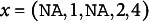 and 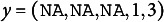 are not complete and not in the same group, however if we project them to the dimensions 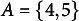 , 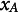 and 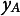 are complete in this lower-dimensional space.

**Figure 1:**
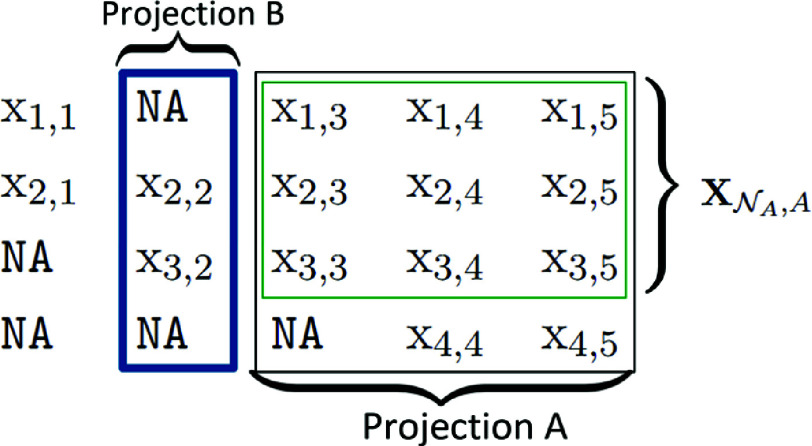
Illustration of the projections *A* and *B* in an example with 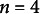 and 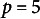 . In a first step, a projection 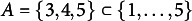 is drawn. The fully observed points on *A* form 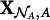 , as indicated in green. In a second step, a projection 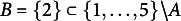 is drawn, as indicated in blue. The patterns in projection *B* then determine the labels assigned to the observations in 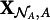 . In this case, we obtain two different class labels: the first observation has one label, and the second and third observations share another common label.

Additionally, to circumvent the problem of many groups with only a few members, we assign new *grouping or class labels* to all observations in 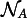 . To do so, we consider the set of projections 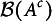 , which is defined as the power set of 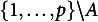 . The set 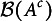 is never empty since 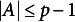 . For a given projection 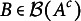 , we project all observations with index in 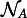 to *B* and form new collapsed missingness pattern groups 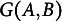 , where 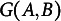 is the set of labels corresponding to distinct missingness patterns among observations with index in 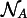 projected to *B*. This is solely done to determine the grouping or class labels of observations with index in 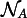 . If two observations with index in 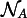 are in the same overall missingness pattern group 
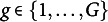
 , they also end up in the same collapsed group. The other direction is not true, that is the number of collapsed groups 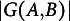 is at most as large as the initial number of distinct groups *G* among the observations with index in 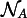 . Considering again 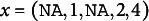 and 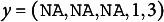 , if 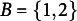 , then observations *x* and *y* are not in the same missingness pattern group. However, if 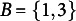 , we assign the same class label to *x* and *y*. Thus, given the projection *A*, we obtain a set of fully observed observations 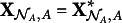 , and given the projection *B* we assign to them the 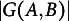 different class labels. Figure 1 provides a schematic illustration of projections *A* and *B* on a more complicated example with four observations, each corresponding to a different pattern (i.e., 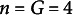 ). According to 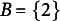 , the first observation in 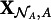 obtains one collapsed class label, whereas the second and third observation obtain another, common label, resulting in 
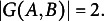

We are now equipped to formulate our one v.s. all-others approach with the hypothesis testing problem (3)

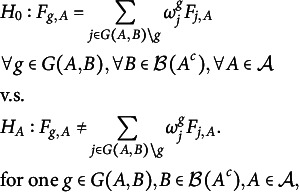

where 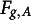 is the joint distribution of the observations of class *g* with index in 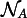 and the groups 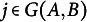 are jointly determined by *A* and *B*. Thus, we compare the distribution of the observed part with respect to *A* of one group *g* with the mixture of the observed parts of the rest of the groups. The weights 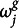 are nonnegative, sum to 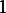 , and are proportional to the respective fraction of observations in class *j*.

Example 1. To give some intuition about the hypothesis testing problem (3), we relate it to the hypothesis testing problem (2) with the help of the example of Figure 1. In this example, each observation 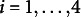 has a different pattern and can thus be seen as a draw from a distribution 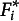 . We first assume that the null hypothesis of (3) holds and show, as an example, that this implies 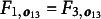 . Since the null hypothesis of (3) refers to all 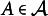 , it also includes 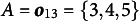 , which is what we consider in Figure 1. While we are only interested in 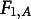 and 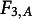 , taking 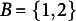 the observations in 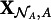 come from the three distributions 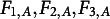 . Due to (3) it holds that (4)

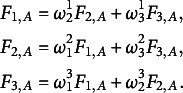

Some algebra shows that equation system (4) is equivalent to 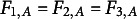 , which in particular means 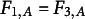 , that we wanted to show. While we took 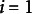 and 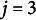 as an example matching Figure 1, we cycle through all 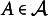 in (3) and thus 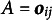 for all patterns 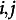 eventually. We now assume that the null hypothesis of (2) is true and consider again 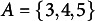 as an example. Since we only look at the fully observed observations in 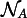 in (3), i.e., leave out the fourth point, we again deal with the three distributions 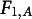 , 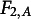 , 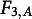 . Moreover, by construction, 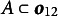 and 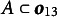 (even 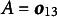 in this case). Thus, 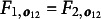 and 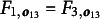 , implied by the null hypothesis of (2), means that 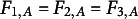 , which implies (4). Again this might seem constructed, but since by definition, (3) only considers the distributions 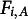 and 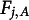 of fully observed points on *A*, it will always hold that 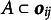 .

We make note of an abuse of notation in (3), as the group *g* in 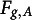 only corresponds to the same index of 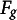 in (2), if 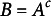 , as can be seen in the example of Figure 1: If 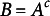 , the three observations in 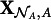 are drawn from 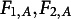 , and 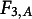 , respectively. However, if 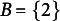 , then observations two and three are now assumed to be drawn from a single distribution, which corresponds to a mixture of 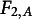 and 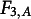 .

In short, the null hypothesis of (3) implies the null hypothesis of (2) because for 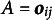 , observations coming from 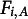 and 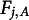 are contained in 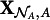 . Vice-versa, the null hypothesis of (2) implies the null hypothesis of (3) because *A* is nested in 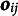 for all 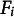 and 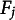 considered on *A*. This actually sketches the proof of the following result:

Proposition 1. Hypothesis testing problem (3) is equivalent to (2).

Tackling hypothesis testing problem (3) would be rather inefficient since we might test many times the same hypothesis when cycling through all 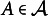 and 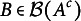 . However, the idea is that *A* and *B* will only be random draws from 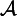 and 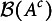 . This is discussed in the next section.

## 4 MCAR test through classification

In this section we introduce the classification-based statistic of our test and detail the implementation of our permutation approach, permuting the rows of the missingness matrix 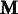 , to obtain a valid test.

### 4.1 Test statistic *U*

Let us fix a projection 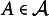 and corresponding projection 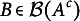 . We denote the induced collapsed class labels based on projections *A* and *B* by 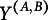 , by 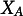 the projection of the random vector *X* on *A* and correspondingly by 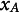 the projection on *A* of observation *x* in 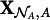 . Furthermore, we define for each 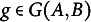 and *x* in 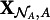 the following quantities: 

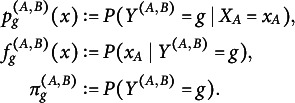

Let us fix 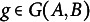 as well. We reformulate the hypothesis testing problem (3): (5)

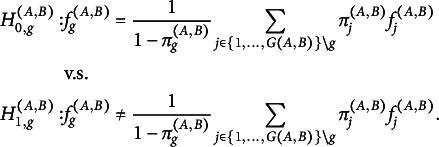



Let 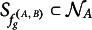 denote the indices of observations in 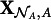 that belong to class *g*. For each missingness pattern *g*, we now define the following statistic in analogy to Cai et al. (2020), (6)

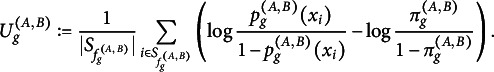



This statistic is motivated by the following claim:

Lemma 1. The logarithm of the density ratio for testing (5) is given by 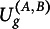 .

The main motivation for the form of this test-statistic is that one can use the same arguments as in Cai et al. (2020, Proposition 1) to show that a test based on 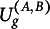 will have the highest power among all tests for (5), according to the Neyman–Pearson Lemma. In addition, the test statistic converges to the Kullback-Leibler Divergence between 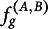 and the mixture of the other densities, motivating the name of our MCAR test. A high value of KL-Divergence indicates that the distributions of two samples deviate strongly from each other.

Lemma 2.

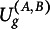
 converges in probability to the Kullback-Leibler Divergence between 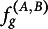 and the mixture of the other densities: 

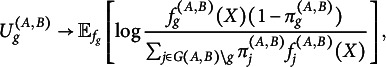

as 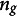 and 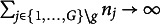 and 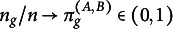 .

Since the statistic 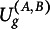 is evaluated only on cases 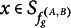 , it holds that 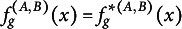 and 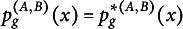 . This means that the projected complete and incomplete distributions coincide on the projected complete samples. Thus we are indeed asymptotically measuring the Kullback-Leibler Divergence between 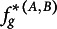 and the mixture of the other densities.

Since there might be only very few observations for a single class *g*, we symmetrize the KL-Divergence. That is, we use the samples of all classes to evaluate the KL-Divergence and not only the samples of class *g*. Let 
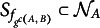
 denote the indices of observations in 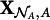 that belong to all other classes 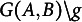 . For each missingness pattern *g*, we will use, in the following, the difference between two of the above statistics, namely (7)

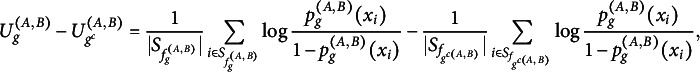

where the terms including the class probabilities 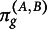 cancel out. This difference converges to the symmetrized KL-Divergence between the mixture of 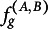 and the remaining classes and is more sample efficient than only using 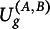 . The test statistic for fixed 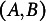 is then given by 

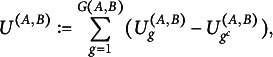

and the final test statistic is defined as (8)

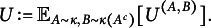



### 4.2 Practical estimation of *U*

We estimate 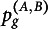 with a multiclass-classifier, yielding 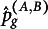 . Plugging-in this quantity into (7) yields 
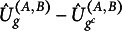
 . We then estimate 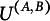 by 

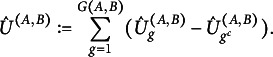

Finally, we estimate *U* by (9)

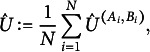

where *N* is the number of draws of pairs of projections 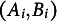 , 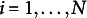 , with 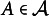 according to a distribution 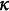 and 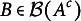 according to a distribution 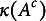 .

Our chosen multiclass classifier is Random Forest (Breiman, 2001; Breiman et al., 1984), more specifically, the probability forest of Malley et al. (2011). That is, for each of the *N* projections, we fit a Random Forest with a specified number of trees, a parameter called 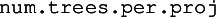 . Thus, for each tree (or group of trees), a random subset of variables and labels is chosen based on which the test statistic is computed. In each tree, we set 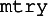 to the full dimension of the projection to not have an additional subsampling effect. This approach aligns naturally with the construction of Random Forest, as the overall approach might be seen as one aggregated Random Forest, which restricts the variables in each tree or group of trees to a random subset of variables. We finally use the OOB-samples for predicting 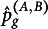 .

The question remains how to sample the sets 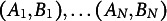 at random. Our chosen approach is quite simple: we first randomly sample a number of dimensions 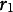 by drawing uniformly from 
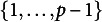
 . We then draw 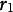 values without replacement from 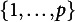 to obtain *A*. Similarly, we randomly draw a value 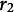 from 
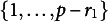
 and then draw 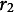 values without replacement from 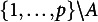 to obtain *B*. We then consider 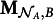 , i.e., all patterns for the fully observed observations in *A* projected to *B*, and build the labels 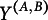 based on the patterns in this matrix. This simple approach is used as a default, but one could also employ a more data-adaptive subsampling. In our algorithm, we might restrict the number of collapsed classes by selecting *B* corresponding to *A* accordingly. The parameter indicating the maximal number of collapsed classes allowed is given by size.resp.set. If set to 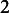 , we reduce the multi-class problem to a two-class problem. In Algorithm 1 we provide the pseudo-code for the estimation of 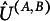 .

To ensure that the level is kept by a test based on the statistic 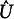 for any choice of 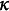 and 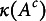 , we use a permutation approach, as detailed next.

### 4.3 Permutation test

To ensure the correct level, we follow a permutation approach. Informally speaking, the permutation approach works in this context if the testing procedure can be replicated in exactly the same way on the randomly permuted class labels. This is not completely trivial in this case, as the labels are defined in each projection via the missingness matrix 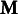 . It can be shown numerically that permuting the labels at the level of the projection does not conserve the level, as this is blind to the correspondence between the projections across the permutations.

**Figure:**
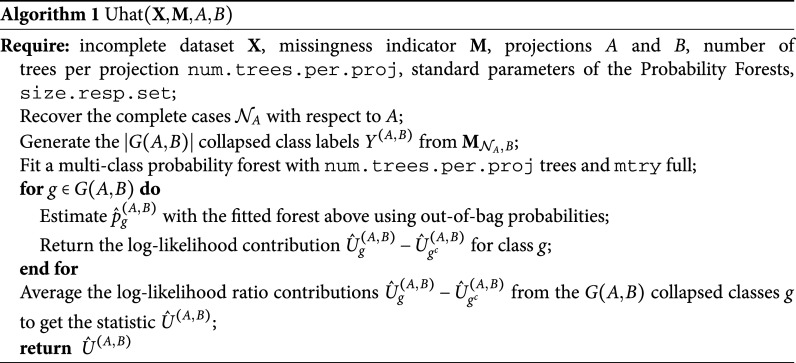

The key to the correct permutation approach is to permute the rows of 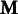 . That is, for *L* permutations 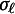 , 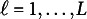 , we obtain *L* matrices 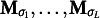 with only the rows permuted. Then we proceed as above: We sample 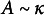 , 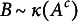 and for each permutation of rows 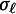 , 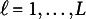 , we calculate 
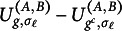
 as in (7). Using 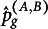 instead of 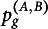 this results in 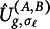 and in the statistic 

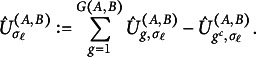

We note that we do not need to refit the forest for this permutation approach to work. Instead, we can directly use 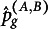 from the original Random Forest that we fitted on the original 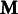 .

Finally, we calculate the empirical distribution of the test-statistic under the null, by calculating for 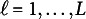 , (10)

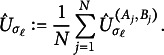

The *p*-value of the test is then obtained as usual by (11)

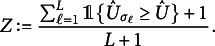



Then it follows from standard theory on permutation tests that *Z* is a valid *p*-value:

Proposition 2. Under 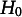 in (1), and *Z* as defined in (11), it holds for all 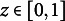 that (12)

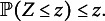

Algorithm 2 summarizes the testing procedure.

**Figure:**
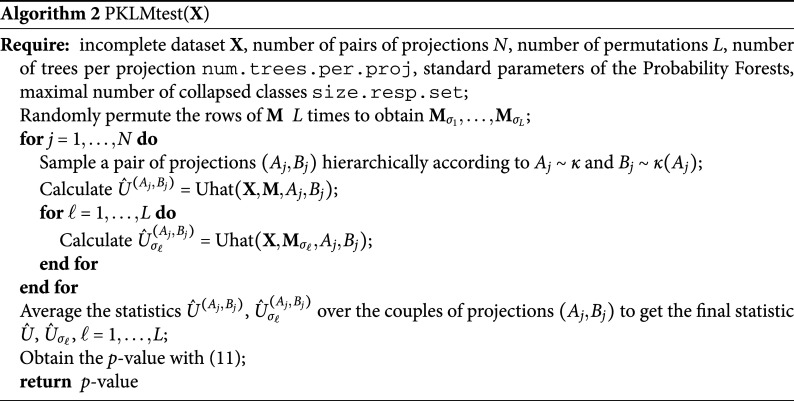

## 5 Empirical validation

In this section, we empirically showcase the power of our test in comparison to recent competitors on both simulated and real data. The simulation setting is set up along the lines of Jamshidian and Jalal (2010) and Li and Yu (2015) with a common MAR mechanism. For the real datasets, we also add a random MAR generation through the function ampute of the R-package mice, see e.g., Schouten et al. (2018). As we did throughout the paper, we refer to our test as “PKLM,” the test of Li and Yu (2015) as “Q,” the test of Little (1988) as “Little,” and finally the one of Jamshidian and Jalal (2010) as “JJ”. The Little-test is computed with the R-package naniar (Tierney & Cook, 2023), while the JJ-test uses the code of the R-package MissMech (Jamshidian et al., 2014). Finally, the code for the Q-test was kindly provided to us by the authors.

### 5.1 Simulated data

We vary the sample size *n*, the number of dimensions *p*, and the number of complete observations, which we denote by *r*. Cases 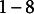 describe the following different data distributions, similarly as in Li and Yu (2015) and in Jamshidian and Jalal (2010): Throughout, 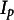 is a covariance matrix with diagonal elements 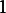 and off-diagonal elements 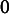 while 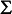 is a covariance matrix with diagonal elements 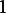 and off-diagonal elements 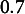 :

1. A standard multivariate normal distribution with mean 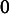 and covariance 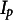 ,
2. a correlated multivariate normal distribution with mean 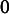 and covariance 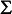 ,
3. a multivariate *t*-distribution with mean 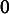 , covariance 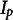 , and degree of freedom 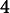 ,
4. a correlated multivariate *t*-distribution with mean 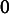 , covariance 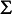 , and degree of freedom 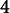 ,
5. a multivariate uniform distribution which has independent uniform 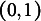 marginal distributions,
6. a correlated multivariate uniform distribution obtained by multiplying 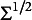 to the multivariate uniform distribution in 5,
7. a multivariate distribution obtained by generating 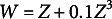 , where *Z* is from the standard multivariate normal distribution,
8. a multivariate Weibull distribution which has independent Weibull marginal distribution, and each Weibull marginal distribution has scale parameter 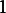 and shape parameter 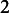 .

The above implements the fully observed 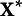 . To compute the type-I error, we then simulate the MCAR mechanism where each value in the *p* columns of the missingness matrix 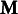 has a probability of 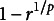 being one and is otherwise zero. To compute the power, we simulate the MAR mechanism following the description in Li and Yu (2015): We generate 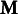 such that the first column consists only of zeros so that the first variable is fully observed. Further, each value in the remaining 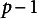 columns has a probability of 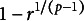 being one, while the rest is zero. This results, on average, in *r* rows in 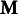 with only zeros, and thus in *r* fully observed rows in 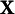 . Next, we sort the rows of 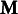 into two groups, those that will be fully observed (complete group) and those that will have at least one missing value (missing group). So far, the generation is still MCAR. However now, for each row 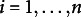 we compare 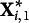 with the mean of 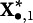 , denoted by 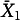 . If 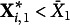 , the corresponding row *i* is placed into the complete group with probability 1/6, and with probability 5/6 into the missing group. That is, with probability 1/6, the row *i* is paired with a row in 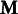 from the complete group, and with probability 5/6, it is paired with a row from the missing group. Thus, in this case it is 5 times more likely that the row is placed in the missing group. On the other hand, if 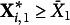 the situation reverses, and row *i* is 5 times more likely to be associated with a row in 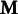 from the complete group. Assigning the rows of 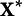 successively to the rows of 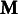 like this results in 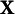 with MAR missingness.

Each experiment was rerun 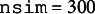 times to compute type-I error and power. We used the following default hyperparameter setting for the computation of our PKLM-test: number of permutations 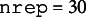 , number of projections 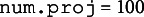 , minimal node size in a tree 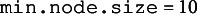 , number of fitted trees per projection 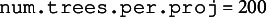 , and maximal number of collapsed classes allowed in a projection 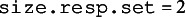 . We note that the choice of these hyperparameters is intriguingly simple: besides 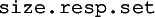 , it holds that “higher values are better”. Thus, as with RF in general, it is mostly a question of computational resources determining how large the values can be chosen. This is especially true for the number of trees for each forest, which should be relatively high in order to minimize additional randomness. We found 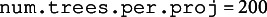 to be a good compromise between speed and accuracy. As the level is guaranteed for any number of permutations, and we desired a choice of hyperparameters that would work for 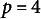 as well as 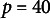 , we chose the number of permutations low 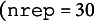 ), but the number of projections relatively high 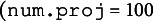 ). The only “difficult” parameter to set is 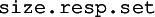 , as there appears to be some loss in accuracy when the number of classes is larger than two. We thus found that 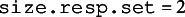 , generating two classes, works well in a wide range of examples.

**Table 3:** Simulated power and type-I error of PKLM, Q, Little and JJ for 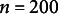 , 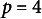 and 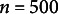 , 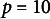

				Power				Type-I error			
*n*	*p*	*r*	Case	PKLM	*Q*	Little	JJ	PKLM	*Q*	Little	JJ
200	4	0.65	1	0.73	**0.98**	0.98	0.12	**0.03**	**0.03**	0.06	**0.04**
			2	**0.93**	1.00	0.96	0.04	**0.03**	0.06	0.06	**0.05**
			3	0.81	**0.94**	0.92	0.05	**0.03**	**0.02**	**0.04**	0.08
			4	0.89	**0.97**	0.91	0.05	**0.01**	**0.03**	**0.05**	**0.05**
			5	0.79	**1.00**	**1.00**	0.19	**0.03**	**0.04**	**0.04**	0.06
			6	0.90	**1.00**	0.99	0.20	**0.03**	**0.04**	**0.03**	0.13
			7	**0.80**	0.93	0.95	0.04	**0.04**	0.06	0.09	0.08
			8	0.72	**0.92**	0.90	0.26	**0.03**	**0.05**	**0.04**	0.08
200	4	0.35	1	0.79	**0.98**	0.97	0.04	**0.03**	**0.04**	**0.04**	0.13
			2	0.87	**0.98**	0.97	0.08	**0.03**	**0.03**	**0.03**	0.08
			3	0.82	**0.97**	0.90	0.16	**0.03**	**0.03**	0.06	0.12
			4	0.87	**0.99**	0.92	0.10	**0.03**	**0.02**	0.08	0.11
			5	0.79	**0.99**	**0.99**	0.10	**0.04**	**0.05**	**0.05**	0.08
			6	0.80	**1.00**	0.97	0.12	**0.03**	**0.04**	0.06	0.11
			7	0.79	**0.98**	0.92	0.09	**0.03**	**0.05**	0.07	0.06
			8	0.83	**0.99**	0.99	0.10	**0.05**	**0.05**	0.06	**0.05**
200	4	0.10	1	0.30	**0.40**	0.26	0.20	0.06	**0.03**	**0.05**	0.22
			2	**0.35**	0.50	0.27	0.12	**0.03**	0.10	**0.05**	0.18
			3	0.25	**0.29**	0.18	0.21	**0.04**	**0.01**	**0.04**	0.24
			4	0.37	**0.42**	0.17	0.19	**0.03**	**0.03**	**0.03**	0.17
			5	0.27	**0.51**	0.33	0.26	**0.05**	**0.02**	**0.05**	0.20
			6	0.31	**0.40**	0.27	0.24	**0.03**	**0.03**	**0.04**	0.17
			7	0.26	**0.42**	0.22	0.20	**0.04**	**0.04**	0.09	0.31
			8	0.31	**0.39**	0.32	0.23	**0.03**	**0.03**	**0.04**	0.18
500	10	0.65	1	**0.93**	0.89	0.88	0.09	**0.05**	0.06	0.06	**0.05**
			2	**0.99**	1.00	0.84	0.08	**0.02**	0.06	**0.05**	**0.05**
			3	**0.85**	0.26	0.61	0.12	**0.02**	**0.05**	0.18	0.10
			4	**0.99**	0.96	0.60	0.10	**0.04**	0.06	0.19	0.12
			5	0.89	**0.98**	0.96	0.16	**0.04**	**0.05**	**0.03**	0.10
			6	**0.99**	1.00	0.91	0.15	**0.04**	0.07	**0.02**	0.13
			7	**0.90**	0.61	0.68	0.09	**0.02**	0.07	0.12	0.07
			8	**0.79**	0.76	0.76	0.18	**0.03**	**0.04**	**0.05**	0.09
500	10	0.35	1	**0.89**	0.74	0.66	0.07	**0.02**	**0.02**	**0.02**	0.08
			2	**0.99**	0.99	0.69	0.09	**0.03**	0.06	**0.03**	0.11
			3	**0.88**	0.33	0.51	0.14	**0.04**	**0.05**	0.18	0.11
			4	**0.98**	0.91	0.48	0.12	**0.04**	0.08	0.20	0.10
			5	**0.91**	0.92	0.83	0.12	**0.04**	0.06	**0.04**	0.12
			6	**0.98**	1.00	0.75	0.09	**0.03**	0.08	**0.04**	0.11
			7	**0.89**	0.46	0.52	0.05	**0.03**	**0.03**	0.08	0.11
			8	**0.92**	0.78	0.74	0.10	**0.05**	0.06	0.06	0.07
500	10	0.10	1	**0.31**	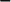	0.06	0.12	**0.02**	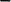	**0.03**	0.10
			2	**0.45**	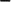	0.07	0.12	**0.03**	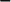	**0.03**	0.07
			3	**0.34**	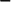	0.18	0.16	**0.03**	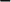	0.19	0.14
			4	**0.45**	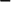	0.20	0.16	**0.02**	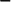	0.22	0.11
			5	0.33	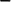	**0.04**	0.12	0.06	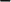	**0.02**	0.14
			6	**0.45**	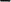	0.03	0.08	**0.05**	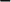	**0.01**	0.12
			7	**0.34**	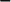	0.12	0.09	**0.05**	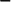	0.09	0.15
			8	**0.34**	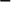	0.04	0.16	**0.03**	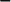	**0.05**	0.13

*Note*: We use 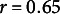 , 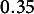 and 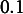 . Cases 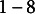 describe different data distributions. The experiments were repeated 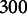 times and the parameter setting for PKLM described above was used.

As mentioned throughout the paper, the Q-test could not be calculated for a large range of settings.^1^ In particular, computation times were infeasible for the setting 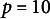 and 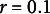 and for any configuration with 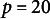 or 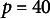 . For the setting 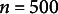 , 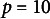 , and 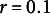 for instance, one test for case 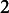 took around 20 minutes to finish, implying an approximate overall computation time of 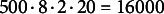 minutes or approximately 110 24-hour days. This despite the fact that the R-code of the Q-test we received was well implemented. In the upcoming Tables 3 and 4 of results, we always used the nominal level of 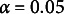 . We boldfaced the results for each row in the tables in the following manner: Whenever the type *I* error of a test is below or equal to 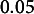 and the test has the best power, it will be boldfaced. If this is true for more than one test, they are all boldfaced. Additionally, we boldfaced all the type-*I* errors that are below or equal to the nominal level 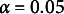 to indicate which tests holds the level on average in the given settings.

In the simulation set-up of 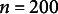 and 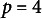 , the Q-test is very powerful, while keeping the nominal level. The PKLM-test is rarely the most powerful here; however, the power of the PKLM-test is often relatively close to the best power. As an example, in case 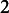 for 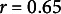 , the Q-test has a power of 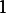 while the PKLM-test has a power 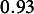 , with both keeping the nominal level 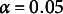 .

In the set-up of 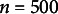 and 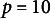 , the overall picture changes. The PKLM-test is in all but two of the 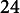 cases the most powerful test, sometimes leaving the second-best test quite far behind. As an example, in case 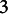 for 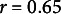 , the PKLM-test has a power of 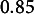 while the Q- and the Little-test exhibit a power of 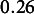 and 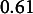 , respectively. While the Little- and the JJ-test often show inflated levels, this is never a problem for the valid PKLM-test.

In the simulation set-up of 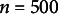 and 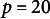 , it appears as if the Little-test is a strong competitor. But this is only until one considers its type-I error. Though to a much lesser degree than the JJ-test, the type-I error is often heavily larger than the nominal level. Considering for instance case 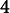 , the power of the Little-test is even slightly less than its actual type-I error for 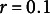 . In case 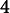 with 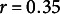 , our test displays a power of 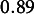 and keeps the level, while the Little-test only has a power of 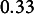 despite having a grossly inflated type-I error. All of these problems are worsened for the JJ-test, which often displays an inflated type-I error in almost all cases and simulation set-ups. A similar story plays out in the case 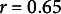 .

**Table 4:** Simulated power and type-I error of PKLM, Q, Little, and JJ for 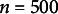 , 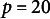 , 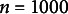 , and 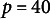

				Power				Type-I error			
*n*	*p*	*r*	Case	PKLM	*Q*	Little	JJ	PKLM	*Q*	Little	JJ
500	20	0.65	1	**0.39**	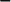	0.36	0.06	**0.02**	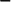	**0.05**	0.09
			2	**0.91**	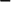	0.48	0.08	**0.03**	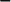	**0.05**	0.10
			3	**0.33**	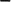	0.49	0.20	**0.03**	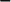	0.24	0.11
			4	**0.90**	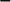	0.40	0.14	**0.04**	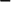	0.22	0.11
			5	0.32	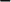	**0.64**	0.14	**0.04**	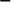	**0.04**	0.08
			6	**0.93**	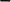	0.39	0.25	**0.04**	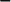	**0.01**	0.09
			7	**0.33**	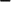	0.37	0.07	**0.03**	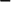	0.09	0.10
			8	**0.23**	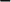	0.25	0.14	**0.04**	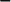	0.06	**0.03**
500	20	0.35	1	**0.45**	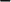	0.22	0.08	**0.03**	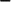	**0.04**	0.09
			2	**0.90**	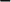	0.22	0.09	**0.03**	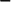	**0.04**	0.08
			3	**0.43**	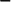	0.35	0.18	**0.02**	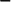	0.34	0.12
			4	**0.89**	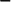	0.33	0.20	**0.03**	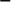	0.31	0.15
			5	**0.46**	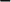	0.24	0.09	**0.02**	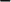	**0.02**	0.12
			6	**0.91**	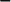	0.14	0.14	**0.02**	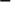	**0.03**	0.10
			7	**0.41**	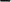	0.22	0.11	**0.02**	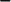	0.11	0.10
			8	**0.52**	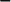	0.18	0.08	**0.03**	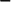	**0.04**	0.07
500	20	0.10	1	**0.13**	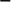	0.00	0.14	**0.03**	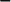	**0.00**	0.10
			2	**0.24**	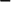	0.01	0.14	**0.04**	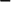	**0.01**	0.12
			3	0.08	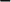	0.21	0.16	0.06	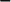	0.22	0.10
			4	**0.26**	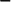	0.27	0.08	**0.04**	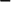	0.31	0.13
			5	**0.12**	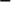	0.00	0.10	**0.03**	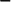	**0.00**	0.19
			6	**0.19**	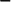	0.00	0.11	**0.05**	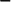	**0.00**	0.18
			7	**0.07**	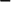	0.08	0.12	**0.04**	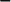	0.07	0.12
			8	**0.07**	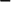	0.02	0.11	**0.04**	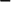	**0.00**	0.16
1000	40	0.65	1	**0.20**	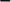	0.00	0.09	**0.05**	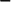	**0.00**	0.15
			2	**0.95**	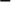	0.00	0.12	**0.03**	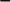	**0.00**	0.14
			3	**0.23**	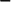	0.00	0.29	**0.02**	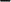	**0.00**	0.17
			4	**0.94**	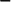	0.00	0.26	**0.05**	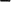	**0.00**	0.17
			5	**0.16**	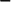	0.00	0.30	**0.02**	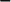	**0.00**	0.19
			6	**0.97**	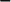	0.00	0.26	**0.02**	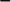	**0.00**	0.19
			7	**0.23**	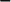	0.00	0.11	**0.02**	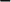	**0.00**	0.10
			8	**0.13**	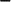	0.00	0.17	**0.03**	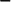	**0.00**	0.12
1000	40	0.35	1	**0.35**	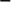	0.00	0.12	**0.02**	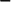	**0.00**	0.11
			2	**0.97**	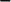	0.00	0.13	**0.05**	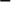	**0.00**	0.10
			3	**0.37**	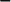	0.00	0.30	**0.03**	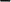	**0.00**	0.30
			4	**0.96**	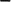	0.00	0.33	**0.04**	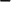	**0.00**	0.27
			5	**0.32**	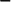	0.00	0.14	**0.04**	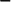	**0.00**	0.11
			6	**0.98**	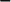	0.00	0.16	**0.03**	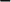	**0.00**	0.10
			7	**0.36**	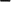	0.00	0.11	**0.02**	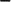	**0.00**	0.08
			8	**0.30**	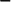	0.00	0.16	**0.02**	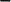	**0.00**	0.10
1000	40	0.10	1	**0.08**	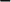	0.00	0.15	**0.02**	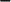	**0.00**	0.12
			2	**0.32**	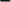	0.00	0.12	**0.02**	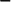	**0.00**	0.10
			3	**0.06**	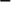	0.00	0.13	**0.05**	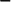	**0.00**	0.20
			4	**0.25**	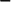	0.00	0.25	**0.03**	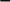	**0.00**	0.28
			5	**0.08**	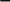	0.00	0.11	**0.03**	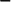	**0.00**	0.09
			6	**0.27**	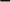	0.00	0.09	**0.04**	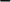	**0.00**	0.11
			7	**0.07**	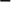	0.00	0.16	**0.03**	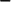	**0.00**	0.13
			8	**0.07**	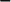	0.00	0.15	**0.04**	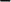	**0.00**	0.08

*Note*: We use 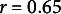 , 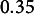 , and 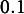 . Cases 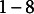 describe different data distributions. The experiments were repeated 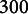 times and the parameter setting for PKLM described above was used.

**Table 5:** Simulated power and level of PKLM, Q, Little and JJ for 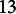 real datasets

			Power				Type-I error			
Dataset	*n*	*p*	PKLM	*Q*	Little	JJ	PKLM	*Q*	Little	JJ
iris	150	4	0.41	**0.91**	0.84	0.27	**0.03**	**0.04**	**0.03**	0.16
blood.transfusion	748	4	0.48	0.97	**1.00**	NA	**0.01**	0.06	**0.04**	NA
airfoil	1503	6	**0.92**	0.13	0.17	0.09	**0.02**	**0.03**	0.06	0.42
seeds	210	7	0.64	**0.74**	0.57	0.24	**0.05**	**0.02**	**0.02**	0.10
yacht	308	7	0.60	0.56	**0.76**	0.24	**0.03**	0.07	**0.05**	0.24
yeast	1484	8	**0.82**	0.52	0.15	0.14	**0.05**	0.06	0.23	0.85
glass	214	9	0.10	0.02	**0.20**	0.20	**0.01**	**0.00**	**0.03**	0.33
concrete.compression	1030	9	0.64	0.48	**0.81**	0.47	**0.04**	**0.04**	**0.05**	0.41
wine.quality.red	1599	11	**0.81**	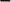	0.72	0.80	**0.04**	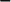	0.15	0.52
wine.quality.white	4898	11	**0.98**	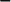	0.96	0.87	**0.04**	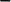	0.10	0.79
planning.relax	182	12	**0.29**	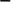	0.20	0.14	**0.00**	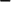	**0.00**	NA
climate.model.crashes	540	19	**0.18**	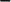	0.22	0.47	**0.00**	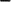	**0.00**	NA
ionosphere	351	32	**0.45**	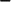	0.97	0.18	**0.00**	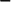	0.06	NA

*Note*: We use 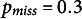 . The experiments were repeated 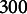 times and the parameter setting for PKLM described above was used. The NAs for some values of the JJ-test indicate that the test was not computable in any of the 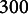 repetitions due to not enough observations in enough usable missingness groups.

Finally, in the simulation set-up of 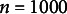 and 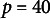 , the power of our test is again much better than that of all other tests. Interestingly, the PKLM-test tends to have higher power when the components of the distribution are not independent, such as in the cases 2, 4, 6, and 8. For example, in case 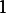 for 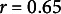 , PKLM has a power of 0.2, while for case 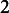 it has a power of 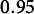 . The main difference between these two cases is the strong positive correlation induced in case 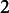 . This pattern repeats: in all correlated examples and for both 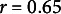 and 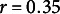 , the PKLM has a power nearing 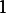 , whereas in the independent versions, the power is closer to the type-I error. Thus, our test is able to use the dependencies in the data to its advantage, at least for 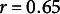 and 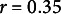 , and can reach a very high power even for comparatively large *p*.

In summary, our test is very competitive even in small dimensions, where the Q-test is very powerful. It leaves behind all other tests by a wide margin as soon as one increases *p*. The Q-test remains strong in these situations as well, but becomes quickly infeasible as either *p* increases or the fraction of complete cases *r* decreases. Crucially, only the PKLM-test and the Q-test are able to consistently keep the nominal level over all experiments, with the Little- and JJ-test showing blatant inflation of the type-I error in many situations. This is the case despite the fact that simply checking the type-I error for a single level 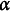 ( 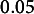 in this case) is far from sufficient to analyse the validity of a *p*-value.

**Figure 2:**
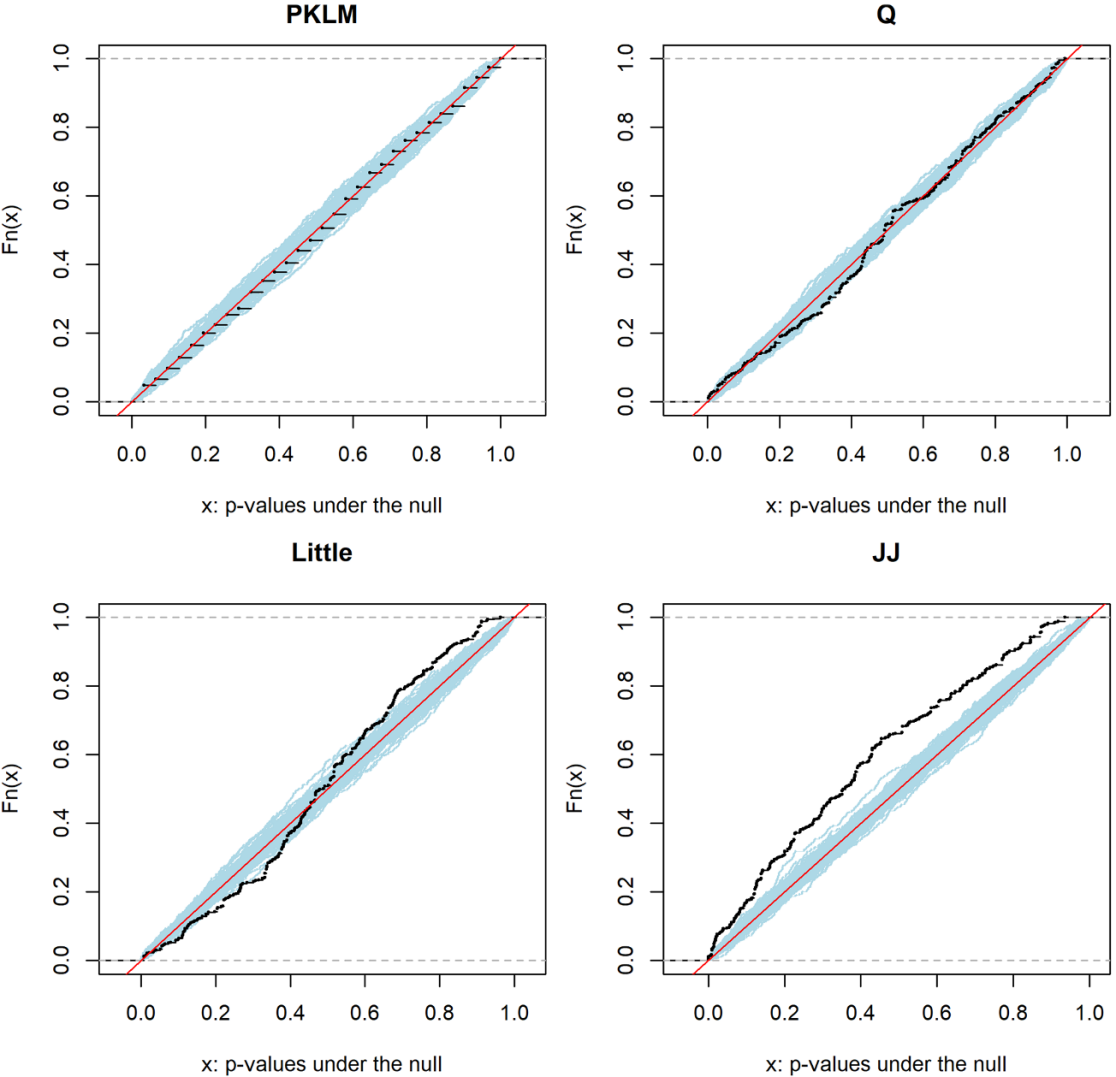
Example plot of cumulative distribution function values of the *p*-values under the null (MCAR) of the four different tests. The simulation set up is 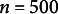 , 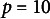 , 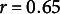 in case 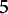 , with 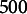 repetitions. The red line is the 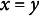 line, while the blue lines show 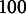 ecdfs of 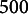 simulated uniform random variables.

As an illustration, we randomly chose one of the above experiments in which the Little-test kept the nominal level, e.g., in the simulation set up 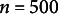 , 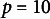 , 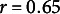 in case 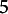 . In Figure 2, we plot the empirical cumulative distribution functions (ecdf) of 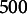 *p*-values under the null (MCAR) of the four different tests. The red line is the 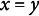 line. In blue we plotted 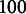 ecdfs of a uniform 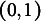 -distribution. As described in Equation (A.6), a valid *p*-value has the property that the corresponding black ecdf values do not lie above the region defined by the blue lines. As Proposition 2 predicts, this is clearly the case for the PKLM-test. That the *p*-values appear rather discrete stems from the fact that we chose a low number of permutations 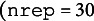 ). The Q-test is sometimes overshooting the red line, though this appears to mostly stem from estimation error. In general, it is remarkable how closely the ecdfs of *p*-values from both the Q- and PKLM-test resemble the ecdf of a uniform sample. The JJ-test appears to consistently have 
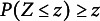
 . The Little-test finally appears to produce a valid *p*-value as long as only values 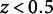 are considered. For 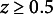 , the the ecdf clearly violates the requirement of a valid *p*-value. If there is no theoretical guarantee, it is thus important to not just check the type-I error at 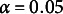 , but to instead consider other levels, e.g., 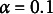 .

### 5.2 Real data

We used 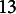 real datasets with varying number of observations *n* and dimensions *p* for further empirical assessment of the PKLM-test and comparison to the other three tests. The datasets are available in the UCI machine learning repository^2^. We preprocessed the data by cancelling factor variables, in order to be able to run all other three tests. However, we kept numerical variables with only few unique values.

For the generation of the NAs, we use an overall probability of missingness of 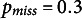 (not to be confused with *r* from the last subsection, denoting the number of complete cases). We used a random MAR generation through the function ampute of the R-package mice. This function can randomly generate realistic MAR mechanisms, see e.g., Schouten et al. (2018). Each experiment was run 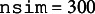 times to compute the type-I error and power. We used the following hyperparameter setting for the computation of our PKLM-test: number of permutations 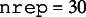 , number of projections 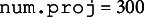 , minimal node size in a tree 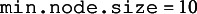 , number of fitted trees per projection 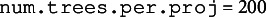 and maximal number of collapsed classes allowed in a projection 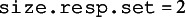 . The results are shown in Table 5. Our test is again very competitive with the best power in 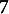 out of 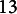 datasets, conditional on valid type-I errors. The Little-test shows also often good performance, though given the problematic level displayed in the previous section, this has to be considered with some care. The Q-test also has relatively high power in the situations where it can be calculated. However, due to computational time we only run the Q-test for 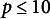 . All in all, we see that the Q-test quickly gets infeasible for large *p* and *n* and the advantage of the PKLM-test strengthens with increasing *p*.

**Figure 3:**
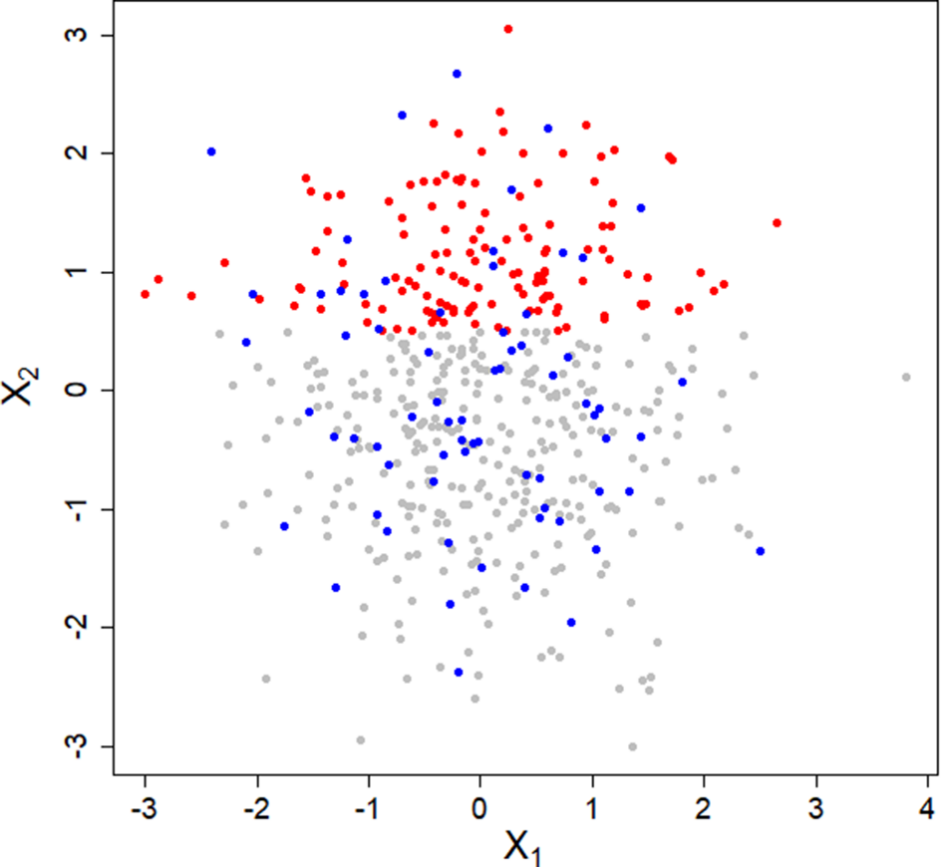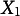 and 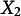 of the fully observed data in the simulated example of Section 6. In red: Points with missing values in 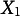 , in blue: points with missing values in 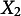 . The blue points are randomly scattered, independently of the value of 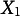 , while in the red points, there is a visible trend toward having more missing values in 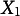 for higher values of variable 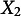 .

## 6 Extension

In addition to the “global test” of MCAR, we can study the effect of single variables: For any given variable 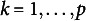 , we can calculate 

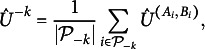

where 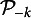 are all pairs of projections 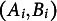 from the *N* randomly chosen ones, with 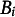 not containing variable *k*. We can use the analogous calculation based on the permuted missingness matrix 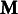




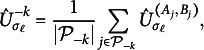

to obtain the *p*-value as in (11). This “partial” *p*-value is valid and corresponds to the effect of removing the patterns induced by variable *k*. Indeed, assume the difference in the distribution of two patterns stems from a variable *j* alone. If 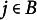 , a perfect classifier will be able to reliably differentiate the two, leading to a high value for 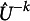 relative to the permutation values. If *j* is not forming the labels, we will not test these two classes against each other and thus not be able spot this difference. As such, we might expect to see a high *p*-value for 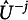 , when variable *j* is removed, but a tendency to low *p*-values for 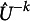 , 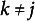 .

We illustrate the usefulness of partial *p*-values with an example. Let 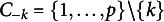 . We assume 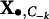 has a MCAR missingness structure, in particular, we simulate below the MCAR mechanism described in Section 5.1 with 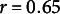 . Let 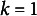 and assume that this first column of observations 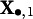 has missingness depending on the observed values of 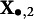 . For instance, each value is missing if the mean of the corresponding row 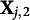 is larger than 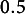 . In this simple example 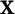 is MAR, but 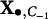 is MCAR. We simulate this example, with 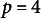 and 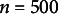 , 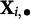 being independent standard Gaussian and the MAR/MCAR mechanism as described above. The first two fully observed components, 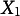 and 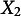 , are shown in Figure 3. As before, we set num.trees.per.proj= 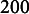 and use 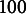 projections. In this example, we are only able to spot any difference when 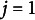 is used to build the labels.

Our test reliably delivers small *p*-values 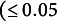 ) for the three partial tests based on projections potentially including variable 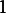 , i.e., sets of projections 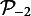 , 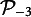 , and 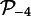 and a high *p*-value for the partial test based on 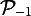 . Thus in this sense, the test detects that the main culprit of the MAR mechanism lies in the first variable.

## 7 Concluding remarks

In this paper, we presented the powerful, flexible and easy-to-use PKLM-test for the MCAR assumption on the missingness mechanism of a dataset. We proved the validity of the *p*-value of the test and showed its power over a wide range of distributions. We also provided an extension allowing to do partial tests, that may shed light on the source of the violation of the MCAR assumption. Naturally, with some slight adaptations the test can be used as a general test of homogeneity of *G* different groups in the sense that it tests whether *G* different groups have the same distribution.

## Supporting information

Spohn et al. supplementary material Spohn et al. supplementary material

## Figures and Tables

**Figure C1:**
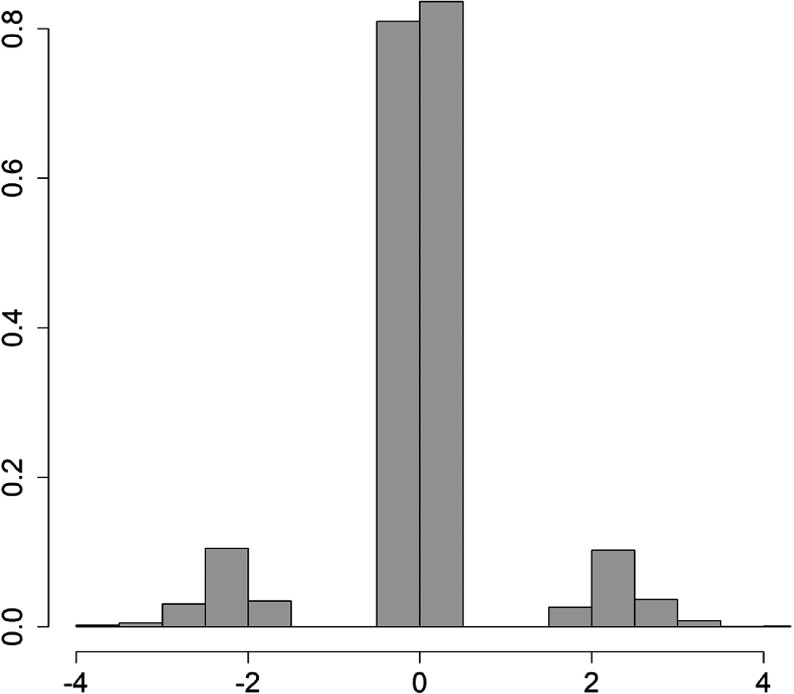
Histogram with relative frequencies of 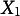 if the corresponding 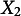 is 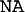 .

## Footnotes

1 The largest number *p* reported in the paper of Li and Yu (2015) is  , while *r* is at least  .

2
https://archive.ics.uci.edu/ml/index.php

## Supplementary material

The supplementary material for this article can be found at https://doi.org/10.1017/psy.2024.14.

## A Proofs

Proposition 1. Hypothesis testing problem (3) is equivalent to (2). Proof. We first show  of (2) implies  of (3). Let  be arbitrary. If they are such that there is only one label, there is nothing to test, so we may assume to have  patterns in  . This means that  for *all* patterns  . This simply follows because, by construction, each of the  patterns in  has the elements in *A* fully observed. But since by assumption for all  ,  and  , this immediately implies that  for all  and thus  . Since  were arbitrary, one direction follows.

We now show that  of (3) implies  of (2). The proof is based on the following claim: Consider *G* arbitrary distribution functions  and weights  ,  such that  for all *j*. Then

(A.1)

We prove the implication by induction: Consider first  . Assuming the LHS of (A.1) and plugging the equation for  into the equation for  , we obtain:

which implies  . Since

we have the equality  and thus  . Plugging this back into the equivalent equation for  , we obtain  . Now assume (A.1) is true for *G* distributions  and we now would like to prove it for  . Assume wlog that the weight of  in the equation of  is nonzero (there will always be at least one such distribution  ). Using the same trick as above, we may plug say the equation for  into  , thereby reducing the number of equations/distributions to *G*. By the induction assumption this implies that  . But immediately this also implies that  and implies (A.1). With this result we can now proof the that  of (3) implies  of (2).

Take two arbitrary groups  and  and take  . To ease notation we just wlog take  and  . Then  contains the dimensions for which patterns  and  have fully observed values. Thus, observations in  contain draws from  and  . Since by assumption

(A.2)

it follows by (A.1), that  for all  and thus in particular,  . Since we will have  for all groups  ,  of (2) holds. Lemma 1. The logarithm of the density ratio for testing (5) is given by  . Proof. Based on the definitions of  ,  and  we obtain by Bayes Rule,

(A.3)

assuming the existence of densities  of distributions  for each  . Following the same steps as in Cai et al. (2020), we get that the logarithm of the (joint) density ratio for testing  vs  of (5), given by

(A.4)

We reformulate the fraction in (A.4) in terms of  , starting from (A.3):

Thus, the inside of the logarithm of (A.4) is given by the following function of  :

Lemma 2.

 converges in probability to the Kullback-Leibler Divergence between  and the mixture of the other densities:

as  and  and  . Proof. From the proof of Lemma 1, we know that  can be rewritten as

(A.5)

Since  and the  are i.i.d., the result follows from the law of large numbers. Proposition 2. Under  in (1), and *Z* as defined in (11), it holds for all  that

(A.6)

Proof. Let  and  be two sets of *N* projections. Let  be all possible permutations of the rows of the missingness matrix  , such that

for  . Note that, since we are only considering fully observed observations for all projections in  ,  , a function of  , is indeed a function of  , while  is a function of  . It also holds that under the null, that is under MCAR, that

(A.7)

This is true because, under MCAR,  and  are independent. Since by the i.i.d. assumption also  for all  and since  ,  are also independent of  , (A.7) follows. As outlined for example in Hemerik and Goeman (2018), this implies that under  ,

Integrating over  , results in (A.6).

## B Additional details and computation times

Here we provide more implementation details, discuss the complexity calculations in Table 1 and show computation times of the different tests in the experiments.

**Numerical truncation.** In order to avoid numerical issues when calculating the density ratio with Expression (6) or the  thereof, if we get predicted probabilities  close to  or  , we apply the following truncation function to  :

**Hyperparameter Selection.** Generally speaking, it holds that “the more the better”, certainly for the parameters *N*, *L* and num.trees.per.proj. As such, the choice of those three parameters depends mostly on the computational power available to the user. For size.resp.set, this is not quite as clear, though we found a value of two to work well in most situations.

**PLKM Test.** We first consider the complexity of one Random Forest, which is in this case

Note that this includes the calculation of  on the test sample through the OOB-error. In total, we do this num.proj times. However, we consider num.tree and num.proj independent of *n* and *p* and thus treating it as constant. In this case, we end up with  . Finally, we need to calculate the statistics *U* and repeat this number of calculations a fixed number of times. This would add a factor  , where again we assume that *B* does not grow with *n* and *p*. As this is neglible compared to  , the complexity is given as  .

**Q-test.** The Q-test compares all groups leading to a complexity of  to compare each group with any other. Additionally, the statistic used is an MMD type, so the complexity is  , where  ,  are the respective group sizes. The group size can be at worst  , which together results in  . The bootstrap on the other hand can also be ignored, as it simply results in a constant factor multiplied to  .

**JJ and Little-test.** Both JJ- and Little-test rely on covariance estimation which scales as  . This gives the  complexity for the Little-test. For the JJ-test one also needs an ordering operation to obtain the test statistics, with complexity  , which results in overall complexity  .

As mentioned above, Table 1 just shows how the complexity scales in *n* and *p* and, in case of our test, treats the number of projections as constants. One might argue that the number of projections should be a function of *p* as well. Similarly, for “small” *p* and small number of groups *G*, the Q-test can be faster than ours. Still the complexities provide a good illustration of how quickly the Q-test can become infeasible, when the number of groups (often a function of *p*) and/or the number of observation increases.

## C Example of Yuan et al. (2018)

Yuan et al. (2018) study settings where group means and variances are approximately equal across missingness patterns, such that MCAR tests based on differences in means and variances, such as the Little-test, have no power. We study one such example here: Let  and  be jointly multivariate normal with correlation zero and let  and

We set  to  if

This corresponds to around  missing values. Figure C1 displays a histogram, plotting all observations of  with  missing for a simulation of  . This corresponds to the MAR example used in Yuan et al. (2018, Section 3) and we refer to their paper for more details.

We simulate the above distribution for  and run our PKLM-test with the same parameters as described in Section 5.1. Though the deviation from MCAR cannot be detected through the first two moments in this example, our test reliably reaches a power of 1.
